# Total neoadjuvant therapy for rectal cancer: where are we now, where do we go?

**DOI:** 10.1016/j.ctro.2026.101254

**Published:** 2026-08-07

**Authors:** Maximilian Fleischmann, Alexander Kristen, Markus Diefenhardt, Emmanouil Fokas, Claus Rödel

**Affiliations:** a Department of Radiotherapy, University Hospital, Goethe-University, Frankfurt, Germany; b Frankfurt Cancer Institute, Frankfurt, Germany; c German Cancer Research Center (DKFZ), Heidelberg, Germany; d Department of Radiation Oncology, Cyberknife and Radiation Therapy, Faculty of Medicine and University Hospital of Cologne, University of Cologne, Cologne, Germany

**Keywords:** Rectal Cancer, Total Neoadjuvant Therapy, Organ Preservation, Chemoradiation, Multimodal

## Abstract

Total neoadjuvant therapy (TNT) refers to the addition of an entire course of preoperative systemic therapy to short-course radiotherapy (SCRT; 5 × 5 Gy) or chemoradiation (CRT), either as induction chemotherapy before CRT or as consolidation therapy after SCRT/CRT, followed by total mesorectal excision (TME) surgery. Randomized trials have demonstrated improved pathological complete response rates and a significant improvement in disease-free survival with TNT compared to neoadjuvant CRT plus surgery with or without adjuvant chemotherapy. TNT is increasingly considered the preferred treatment strategy, especially for patients with MRI-defined high-risk rectal cancer. (such as cT4, cN2, Magnetic Resonance Circumferential Resection Margins Positive (mrCRM+), Extramural Vascular Invasion Positive (EMVI+), lateral lymph node involvement). Key questions remain, including the optimal RT regimen (SCRT vs. CRT), the sequence of treatment components (induction vs. consolidation therapy), the number of chemotherapy cycles, RT-dose escalation strategies, the need of adjuvant chemotherapy after TNT and surgery, and the role of immunotherapy in both mismatch repair–deficient and mismatch repair–proficient (dMMR/pMMR) disease. Due to the higher rates of clinical complete response achieved with TNT, organ-preserving (“watch-and-wait”) strategies are increasingly favored and continue to be refined in ongoing clinical trials (e.g., ACO/ARO/AIO 18.1, JANUS, ENSEMBLE, TRESOR, and STELLAR II).

## Highlights

• TNT improves pCR, DFS, and reduces distant metastases in high-risk rectal cancer.
• Optimal TNT sequencing, RT regimen, and chemotherapy intensity remain unclear.
• TNT increases organ preservation and supports watch-and-wait strategies.

## 1 Background

The management of primary, non-metastatic rectal cancer has evolved from a rigid treatment algorithm to a stratified decision-making process that integrates clinical stage, tumor location, specific features from magnetic resonance imaging (MRI), molecular profile, and patient preference within a multimodal, risk-adapted therapeutic framework.

At the outset, therapeutic stratification in rectal cancer should be guided by molecular profiling. The distinction between mismatch repair–deficient (dMMR)/microsatellite instability-high (MSI-H) or mismatch repair–proficient (pMMR)/microsatellite stable (MSS) disease has immediate therapeutic consequences. In the small subset of dMMR tumors (in the range of 2–5%), single-agent PD-1 blockade has demonstrated remarkable efficacy, achieving surprisingly robust clinical complete responses in a high proportion of patients. These findings have introduced the concept of an immunoablative approach, eliminating the need for conventional therapies such as chemoradiotherapy and radical surgery [1].

Beyond molecular profiling, decision-making in the majority of rectal cancer patients has become a complex, patient-centered process involving a multidisciplinary team to balance oncologic safety, functional outcomes and quality of life (QoL). This includes careful consideration of the impact of multimodal therapies on the risk of a permanent colostomy, organ preservation, and their respective sequelae on bowel, genitourinary, sexual function and other treatment-associated long-term toxicities.

In principle, the following therapeutic options are available for early-stage and locally advanced rectal cancer (LARC): primary surgery (local excision or total mesorectal excision, TME), neoadjuvant chemotherapy, neoadjuvant short-course radiotherapy (SCRT; 5 × 5 Gy), chemoradiotherapy (CRT), total neoadjuvant therapy (TNT) with SCRT or long-course CRT, and organ-preserving strategies without radical surgery in cases of complete or near-complete response to SCRT/CRT/TNT or immunotherapy in patients with dMMR tumors.

This review focuses on recent developments in total neoadjuvant therapy (TNT). TNT integrates additional systemic therapy into the preoperative setting, complementing SCRT or conventionally fractionated CRT with either induction or consolidation chemotherapy, to improve treatment adherence and tolerability while reducing the risk of distant metastases, ultimately translating into improved disease-free (DFS) and overall survival (OS). Of note, randomized trials had previously shown that postoperative chemotherapy after neoadjuvant SCRT/CRT and TME was frequently omitted or delivered at reduced dose and intensity, and did not result in an improvement in disease-free survival [2].

## 2 TNT with SCRT versus standard neoadjuvant CRT

Three phase III trials compared TNT using SCRT plus fluoropyrimidine and oxaliplatin-based chemotherapy versus standard neoadjuvant CRT (Table 1). The RAPIDO trial investigated standard capecitabine-based CRT followed by TME with or without adjuvant FOLFOX4 (12 cycles) or CAPOX (8 cycles) versus SCRT followed by FOLFOX4 (9 cycles) or CAPOX (6 cycles) and surgery in patients with MRI-defined high-risk LARC. Primary endpoint was disease-related treatment failure (DRTF) at 3 years, defined as local and distant failure, colorectal secondary malignancies or treatment-related death. Grade ≥ 3 acute toxicity occurred in 48% of patients in the TNT arm compared with 25% in the CRT arm (*p*-value: not reported), while there was no significant difference in health-related quality of life (HRQOL), bowel function, or late toxicity. Surgical complication rates according to the Clavien–Dindo classification did not differ significantly between the groups. The pathological complete response (pCR) rate was significantly higher in the TNT arm (28.4%) than after CRT (14.3%; *p* < 0.001). After a median follow-up of 4.6 years, the TNT arm showed a significant improvement in the primary endpoint compared with neoadjuvant CRT (3-year DRTF 23.7% vs 30.4%; HR 0.75; *p* = 0.019). In addition, the rate of distant metastases at 3 years was significantly lower in the TNT arm (20.0% vs 26.8%; HR 0.69; *p* = 0.005). In the initial report, the 3-year local recurrence rate after TME was 6.0% following CRT and 8.7% after TNT (HR 1.45; *p* = 0.09) [3]. With longer follow-up, however, the updated 5-year analysis demonstrated a significantly higher rate of locoregional recurrence in the TNT arm than in the CRT arm (10.2% vs 6.1%; *p* = 0.027) [4]. The reasons for the increased rate of locoregional recurrence remain controversial. Beyond the potentially different efficacy of SCRT compared with long-course CRT (lower biologically effective dose (BED) and equivalent dose (EQ.D2) delivered with SCRT), particularly in the MRI-high-risk tumors, it is notable that the quality of the TME specimen was rated significantly poorer after TNT (“complete”: 78% vs 85%; *p* = 0.032). One possible explanation is the longer interval between TNT and surgery (approximately 24 weeks), which may induce more fibrosis and thereby complicate surgical dissection. Moreover, Prata et al. showed that locoregional recurrences following sphincter-preserving surgery occurred in 12.1% after TNT and 4.8% after CRT (HR: 2.60), compared to 8.5% versus 7.5%, respectively, after abdominoperineal resection. Although distal resection margins of ≤10 mm after sphincter-preserving surgery occurred with similar frequency following TNT and CRT (17.5% vs. 22.1%), these close distal margins were associated with a substantially higher cumulative incidence of locoregional recurrence in the TNT group (25.4% vs. 1.8%; HR 15.51), highlighting the importance of baseline information on tumor height and extension to guide surgical strategies. [4], [5].

**Table 1:** Study Characteristics, Patient Population and Outcomes of Included Phase III TNT Trials. Abbreviations: EMVI+ extramural vascular invasion, MRF+ mesorectal fascia, SCRT short course radiotherapy, CRT chemoradiation, FOLFOX fluorouracil, leucovorin, oxaliplatin, CAPOX capecitabine, oxaliplatin, FOLFIRINOX fluorouracil, leucovorin, oxaliplatin and irinotecan TME total mesorectal excision, NOM nonoperative management, DRTF disease-related treatment failure, HR hazard ratio

	RAPIDO		STELLAR		Polish II		PRODIGE 23	
Median Follow-up	5.6 Years		35 Months		7 Years		82 Months	
Patient Population	cT4a/b, EMVI+, cN2, MRF+ or enlarged lateral lymph nodes		cT3–4 or cN+		cT4 or palpable fixed cT3		cT3 “with risk” or cT4	
n	462	450	302	297	261	254	231	230
Intervention	Experimental	Control	Experimental	Control	Experimental	Control	Experimental	Control
	SCRT (5 × 5 Gy) > > FOLFOX4 (9 x) or CAPOX (6 x) > > TME	CRT > > TME > > with or without FOLFOX4 (12 x) or CAPOX (6 x)	SCRT (5 × 5 Gy) > > CAPOX (4 x) > > TME or NOM >> CAPOX (2 x)	CRT > > TME > > CAPOX (6 x)	SCRT (5 × 5 Gy) > > FOLFOX4 (3 x) > > TME	CRT > > TME	FOLFIRINOX (6 x) > > CRT > > TME mFOLFOX (6 x) or Cape (4 x)	CRT > > TME mFOLFOX6 (12 x) or Cape (8 x)
Pathological Complete Response Rate (%)	28.4	14.3	21.8	12.3	16	12	28	12
R0 Resection Rate (%)	90	90	91.5	87.8	77	71	95	94
Acute Toxicity Grade 3–4 (%)	48	25	26.5	12.6	23	21	46	36
Primary Outcome	3-year DRTF 23.7% vs. 30.4% HR 0.75, p = 0.019		3-year DFS (non inferiority) 64.5% vs. 62.3% HR 0.88, p < 0.001		R0 Resection Rate 77% vs. 71% p = 0.07		3-year DFS 76% vs. 69% HR 0.69, *p* = 0.034	
Overall Survival (OS)	5-year OS: 81.7% vs. 80.2% *p* = 0.5		3-year OS: 86.5% vs. 75.1% p = 0.033		8-year OS: 49% vs. 49% HR 0.9; p = 0.38		7-year OS: 81.9% vs. 76.1% *p* = 0.033	
Disease-Free Survival (DFS)	5-year DRTF: 27.8% vs. 34% HR 0.79, p = 0.048		See above		8-year DFS: 43% versus 41% *p* = 0.65		7-year DFS: 67.6% vs. 62.5% *p* = 0.048	
Distant Metastasis-Free Survival (DMFS)	5-year DMFS: 23.0% vs. 30.4% *p* = 0.011		3-year DMFS: 77.1% vs. 75.3% *p* = 0.47		8-year DMFS: 36% vs. 34% *p* = 0.54		7-year DMFS: 20.7% vs. 27.7% *p* = 0.066	
Local Recurrence (LC)	5-year LC: 10.2% vs. 6.1% p = 0.027		3-year LC: 8.4% vs. 11% *p* = 0.46		8-year LC: 35% versus 32% *p* = 0.60		7-year LC: 5.3% vs. 8.1% *p* = 0.38	

In another multicenter, randomized phase III trial from China (STELLAR), a TNT strategy consisting of SCRT followed by 4 cycles of CAPOX, TME or non-operative management (NOM) for patients with a clinical complete response (cCR), followed by 2 additional cycles of CAPOX was compared with conventional capecitabine-based CRT, followed by TME/NOM and 6 cycles of CAPOX. The study was designed as a non-inferiority trial. A total of 599 patients with UICC stage II/III tumors of the mid and lower third of the rectum were enrolled. The primary endpoint was 3-year DFS. The prespecified non-inferiority margin was 11% (CRT 65% vs TNT 54%; HR <1.43). While grade 3–4 acute toxicities driven by the neoadjuvant interventions were significantly more frequent in the TNT arm (26.5% vs. 12.6%, *p* < 0.001), the rates of surgical complications were comparable between the two groups. TNT resulted in significantly higher rates of clinical and pathological complete response compared to conventional CRT (cCR + pCR: 22.5% vs 12.6%; *p* < 0.01). After a median follow-up of 35 months, the 3-year DFS was 64.5% in the TNT arm and 62.3% in the CRT arm (HR 0.883; *p* = 0.001 for non-inferiority). No difference was observed in the rate of local recurrence, however OS was significantly improved in the TNT group (86.5% vs 75.1%; *p* = 0.033). Notably, 60 of 230 patients (26.1%) who underwent surgery in the CRT group did not receive any adjuvant chemotherapy, and only 82/230 (35.7%) completed all planned adjuvant cycles [6].

The POLISH 2 trial provided an extended follow-up after conventionally fractionated, fluorouracil-based CRT (the addition of oxaliplatin during CRT was permitted) versus TNT with SCRT followed by 3 cycles of FOLFOX4 and surgery in patients with cT4 or palpable fixed cT3 disease. The primary endpoint (R0 resection rate) did not differ significantly between the two arms (71% vs 77%; *p* = 0.07). Eight years post-treatment, the authors could not report a significant difference between the two arms regarding local control, DFS and OS [7]. However, the POLISH 2 regimen included only a comparatively short period of three cycles of preoperative FOLFOX4, which may have contributed to the absence of a significant R0 resection rate and long-term DFS or OS benefit.

## 3 TNT with conventionally fractionated CRT versus neoadjuvant CRT without additional chemotherapy

The French PRODIGE 23 trial investigated TNT consisting of 6 cycles induction chemotherapy with leucovorin, fluoruracil, irinotecan and oxaliplatin (FOLFIRINOX), followed by capecitabine-based CRT, TME and three months of adjuvant chemotherapy (capecitabine or FOLFOX) versus standard capecitabine-based CRT followed by TME, and six months of adjuvant chemotherapy with either FOLFOX or capecitabine. The inclusion criteria were less precise than in the RAPIDO trial and included patients with cT3 tumors considered at „*risk of local recurrence*“or cT4 tumors located within 15 cm of the anal verge. The primary endpoint was DFS, and a total of 461 patients were enrolled. Surgical morbidity was comparable between the two groups. TNT resulted in a significantly higher pCR rate (28% vs 12%; *p* < 0.001) and significantly improved DFS (3-year: 75.7% vs 68.5%; HR 0.69; *p* = 0.03) as well as metastasis-free survival (78.8% vs 71.7%; HR 0.64; *p* = 0.02) [8]. In an updated analysis of the PRODIGE 23 trial with a median follow-up of 82 months, the benefit of the TNT regimen remained significant. The 7-year DFS rate was 67.6% in the TNT arm compared with 62.5% in the standard CRT arm (HR 0.80; *p* = 0.048). Consistent with these findings, a restricted mean survival time analysis demonstrated a statistically significant OS benefit of 4.3 months (95% CI, 0.4–8.4 months) in favor of the FOLFIRINOX-based TNT compared with standard CRT [9].

Supporting these findings, a meta-analysis of 11 randomized trials including 3101 patients with UICC stage II/III rectal cancer demonstrated that TNT with CRT and induction/consolidation chemotherapy significantly improved oncologic outcomes compared with standard CRT. TNT was associated with a higher pCR rate (OR 1.95; *p* < 0.05) and significantly improved DFS (HR 0.82; p < 0.05) and metastasis-free survival (HR 0.79; p < 0.05) [10].

## 4 Sequencing TNT — Induction chemotherapy versus consolidation chemotherapy

While both induction and consolidation chemotherapy have demonstrated efficacy, the optimal sequencing of systemic therapy within TNT concepts remains unclear. This question was addressed in the phase II CAO/ARO/AIO-12 trial conducted by the German Rectal Cancer Study Group. In this study, 306 evaluable patients received three cycles of FOLFOX either before (induction) or after (consolidation) fluorouracil/oxaliplatin-based CRT, followed by mandatory TME. The primary endpoint was the pCR rate to identify the superior sequencing strategy within a pick-the-winner design for further evaluation in a phase III study. The hypothesis was that TNT would improve the pCR rate to at least 25% compared to a historical rate of 15%. The observed pCR rate was 17% in the induction arm compared with 25% in the consolidation arm. In addition, treatment compliance to CRT was better with consolidation chemotherapy, while surgical complication rates were similar in both groups [11]. After a median follow-up of 43 months, the long-term oncologic outcomes were nearly identical between the two groups. Therefore, the sequence of CRT followed by consolidation chemotherapy appears more favorable, particularly in the context of downstaging and pCR rates, given the improved local tumor regression observed with this approach [12].

## 5 Critical evaluation of studies on TNT

Taken together, these data support TNT as the preferred treatment strategy to improve DFS in patients with MRI-defined high-risk tumors (best defined in the RAPIDO trial). In principle, TNT can be delivered using either SCRT with 5 × 5 Gy (RAPIDO, STELLAR) or conventional CRT (PRODIGE 23, CAO/ARO/AIO-12), although the optimal approach needs to be defined. If organ preservation is intended, the sequence CRT followed by consolidation chemotherapy should be preferred as demonstrated by the CAO/ARO/AIO-12 and OPRA trial (see below).

The optimal duration of consolidation chemotherapy, however, remains unclear, given the wide variation across studies. In a pooled analysis comparing the OPRA trial (CRT with induction or consolidation chemotherapy administered over approximately 4.5 months), and the CAO/ARO/AIO-12 (with a corresponding duration of the additional systemic therapy of only 1.5 months), no difference in DFS or OS was demonstrated [13]. The Swedish nationwide LARCT-US cohort study demonstrated that TNT consisting of SCRT followed by four cycles of CAPOX achieved oncological outcomes comparable to those reported in the RAPIDO trial, despite including an older patient population with less favorable baseline characteristics. These findings suggest that reducing preoperative chemotherapy to four cycles of CAPOX may not compromise the efficacy of TNT [14]. In cases of delayed tumor regression, a longer interval between SCRT/CRT and response assessment may increase rates of cCR and subsequently organ preservation. Conversely, in non-responsive tumors a longer interval between SCRT/CRT and TME may cause tumor progression, emphasizing the necessity of an additional interim staging, as proposed by the authors of the RAPIDO trial. Moreover, the role of irinotecan within induction or consolidation chemotherapy needs to be determined and validated (PRODIGE 23, JANUS).

A critical consideration of TNT approaches is the significantly increased rate of acute toxicity compared with conventional neoadjuvant SCRT/CRT without additional systemic therapy. Particularly in elderly and frail patients with comorbidities, intensified treatment strategies may not be appropriate, underscoring the need for careful patient selection.

## 6 Organ preservation in patients with clinical complete response after TNT

Multimodality treatment including radiation, chemotherapy and surgery in LARC is associated with substantial morbidity and significant impairments in QoL. These sequelae affect bowel, urinary, and sexual function, as well as body image and mental health, particularly as many patients with distal rectal cancers require a permanent colostomy. With regard to organ preservation after TNT in patients with low rectal tumors and clinical UICC stage II (cT3–4 cN0) or stage III (cT_any_ cN1–2), evidence is provided by the aforementioned randomized phase II OPRA trial from the United States. Comparing 4,5 months of induction chemotherapy with CAPOX or FOLFOX followed by CRT (50 to 56 Gy) combined with either continuous fluorouracil or capecitabine during radiotherapy, or upfront CRT followed by an identical consolidation chemotherapy regimen, the primary endpoint for both groups was DFS, with a comparison to a null hypothesis on the basis of historical data. Secondary endpoints included TME-free survival. A total of 324 patients were randomized to Induction-CRT or CRT-consolidation, 304 were analyzable, and 294 had restaging after TNT. Overall, clinical complete response (cCR) at the first restaging was reported in 125 patients (induction versus consolidation CRT: 55, 44%, versus 70, 56%), and near cCR in 114 (induction versus consolidation CRT: 60, 52.6%, versus 54, 47.4%) [15].

Cumulative toxicities did not differ significantly between the two groups. Similarly, no differences were observed in DFS (3-year: 77% vs 78%) or metastasis-free survival (3-year: 82% vs 84%). However, TME-free survival showed a significant advantage for the sequence of CRT-consolidation (59% vs 43% at 3 years; *p* = 0.007). Organ preservation was 77% after cCR and 40% after near cCR (*p* < 0.001) Overall, local regrowth occurred in 75 of 225 patients (33%) managed with a watch-and-wait strategy. Of these, 62 patients (83%) underwent salvage TME and five (7%) were treated with local excision (six declined surgery, one had tumor progression) [13]. Long-term data with a median follow-up of more than 60 months continue to confirm a significant advantage of this sequence with respect to organ preservation (TME-free survival: 54% vs 39% after induction chemotherapy followed by CRT; *p* = 0.012) [16].

Although TME was mandatory in the CAO/ARO/AIO-12 trial regardless of treatment response, the results are consistent with those of the OPRA trial, confirming the superiority of CRT followed by consolidation chemotherapy over induction chemotherapy with regard to complete response and tumor regression grading. Building on these findings, the subsequent multicenter phase II CAO/ARO/AIO-16 trial adopted a selective watch-and-wait strategy (W&W) for patients with cCR after CRT followed by consolidation chemotherapy. A cCR was achieved in 37% of patients (34/91), while an additional 27% demonstrated a pathological complete response (pCR) after primary TME. Remarkably, a subset of patients with a near cCR at the initial response assessment (*n* = 33/91; week 15–16) converted to a cCR by the subsequent assessment 3 months thereafter (21/33, 64%), emphasizing the importance of an adequate interval between completion of CRT. and (final) response assessment. During follow-up, 16 of 34 (47.1%) patients developed local regrowth after an initial cCR and were treated with salvage TME. The 3-year TME-free survival rate was 21% [17]. The NO-CUT single arm phase II trial included patients with cT3-cT4, cN1–2 rectal cancer and its primary endpoint was 30-month distant relapse-free survival (DRFS) in patients who achieved cCR. In total, 180 patients received four cycles of induction CAPOX chemotherapy followed by capecitabine-based CRT. After a median follow-up of 35 months, the 30-month DRFS was 95% in the non-operative management (NOM) group, compared with 74% in the overall cohort and, hence, deferral of surgery did not compromise oncologic outcome. The rates of cCR and pCR were 26% and 6%, respectively, and local (endorectal) regrowth occurred in 15% of patients in the NOM group [18].

## 7 Ongoing trials on TNT and organ preservation

**Fig. 1:**
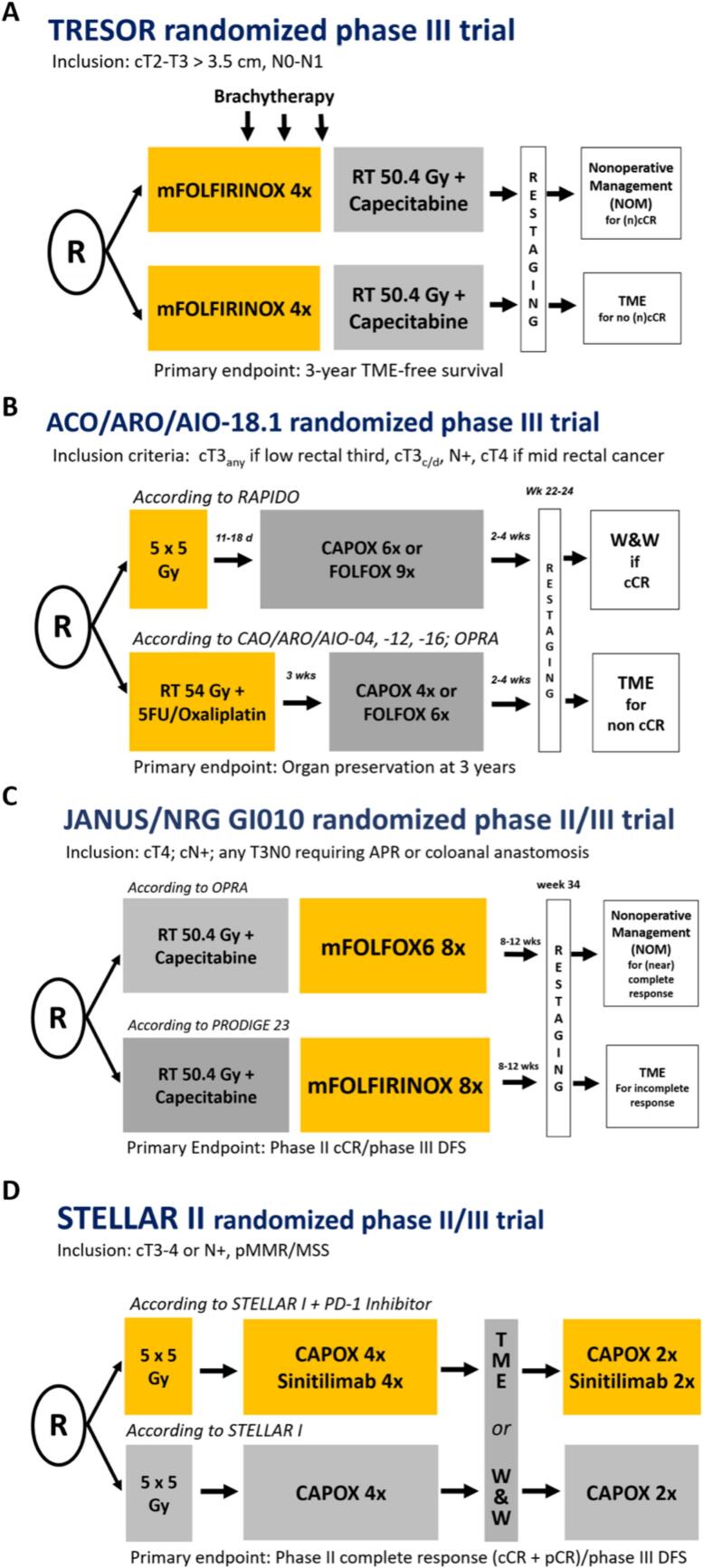
A-D. overview of pending TNT trials. Abbreviations: FOLFOX fluorouracil, leucovorin, oxaliplatin, CAPOX capecitabine, oxaliplatin, FOLFIRINOX fluorouracil, leucovorin, oxaliplatin and irinotecan TME total mesorectal excision, W&W watch & wait, NOM nonoperative management, c/pCR clinical/pathological complete response, DFS disease-free survival.

An alternative strategy to increase organ preservation rates is RT dose escalation. The randomized phase III OPERA trial, investigated the addition of an external beam radiotherapy (EBRT) boost with 9 Gy versus a contact x-ray brachytherapy boost (90 Gy in three fractions) after standard capecitabine-based CRT (45 Gy in 25 fractions) in patients with low cT2–3 rectal cancer. After a median follow-up of 38.2 months, dose escalation with contact X-ray brachytherapy enabled organ preservation in 81% of patients, compared with 59% in the EBRT boost group, and reached up to 97% in patients with tumors smaller than 3 cm in diameter [19]. This concept is now being further investigated in the French randomized phase III TRESOR trial (Fig. 1A), which integrates brachytherapy into a TNT concept. Patients receive 4 cycles of induction mFOLFIRINOX with or without a contact X-ray brachytherapy boost, followed by capecitabine-based CRT. Eligible patients have cT2–3 tumors (>3.5 cm and < 6 cm), cN0–1 disease, located in the low or mid rectum. The primary objective is to achieve a 3-year organ preservation rate of 65% [20].

At present, it remains unclear whether SCRT (5 × 5 Gy), as used in RAPIDO and STELLAR, or CRT with a dose of 50–54 Gy, as applied in PRODIGE 23, OPRA, and CAO/ARO/AIO-12 and -16, should be preferred within TNT strategies—particularly when organ preservation is the primary treatment goal. This question is being addressed in the randomized phase III ACO/ARO/AIO-18.1 trial conducted by the German Rectal Cancer Study Group (Fig. 1B). The primary endpoint is the rate of organ preservation at 3 years. Recruitment was completed in the third quarter of 2023 with a total of 702 patients, and results are expected in early 2027.

The role of irinotecan as part of consolidation chemotherapy (FOLFIRINOX) following initial CRT will be defined by the currently enrolling U.S.-based phase II/III JANUS trial (Fig. 1C) [21]. Eligible patients include those with cT4N0, cT_any_, N+, or cT3 N0 tumors who would require abdominoperineal resection or coloanal anastomosis. The control arm is based on the superior regimen identified in the OPRA trial with CRT followed by doublet chemotherapy with mFOLFOX6 or CAPOX. In the experimental arm, consolidation chemotherapy after CRT consists of triplet chemotherapy with mFOLFIRINOX administered over 3–4 months, analogous to the regimen used in PRODIGE 23. The primary endpoint of the phase II part compares the rate of clinical complete response assessed 8–12 weeks after completion of TNT, while the primary endpoint of the phase III is organ preservation-adapted DFS. The ENSEMBLE trial is an ongoing randomized phase III study in which patients with cT3–4 N0 or cT1-4 N1–2 are assigned to receive SCRT followed by consolidative chemotherapy using either doublet (CAPOX) or triplet chemotherapy with CAPOXIRI (capecitabine, oxaliplatin and irinotecan). The primary endpoint is organ preservation-adapted DFS [22].

## 8 New developments of TNT in pMMR/MSS tumors: inclusion of immune checkpoint inhibitors

Immune checkpoint inhibitors (ICI) show remarkable efficacy in rectal cancer with mismatch repair deficiency (dMMR) [1], but their role in pMMR/MSS disease is limited. Hypothetically, upfront SCRT/CRT could have an immunomodulatory effect and transform “cold” MSS tumors into “hot” immunoreactive tumors with increased antigen presentation, T-cell infiltration, and PD-L1 expression [23]. Recent randomized phase II/III trials of SCRT/CRT/TNT with or without integration of ICIs in pMMR/MSS tumors have demonstrated statistically and clinically meaningful improvements in early efficacy endpoints, such as pCR and cCR rates (Table 2) The Chinese STELLAR II phase II/III trial (Fig. 1D) randomized patients with cT3/4 or cN+ rectal cancer to TNT (SCRT followed by 4 cycles of CAPOX with or without 4 cycles of the PD-1 inhibitor sintilimab during consolidation therapy). The primary endpoint of the phase II part is the cCR/pCR rate, while that of the phase III portion is DFS. Initial results from the randomized phase II part showed a significantly higher CR rate in the TNT + sintilimab arm (45.5% vs. 25.0%; *p* = 0.003), although there was also an increased rate of grade 3/4 acute toxicity (TNT + sintilimab: 34.5% vs. TNT: 19.4%) [31]. Long-term results on DFS are still pending. Meta-analyses of TNT with or without ICI suggest that upfront SCRT with lymphocyte-sparing radiation schedules and the omission of concurrent chemotherapy may exhibit superior efficacy versus CRT [32]. Evidently, more data are needed to refine the optimal sequence of radiation, fractionation, and chemotherapy intensity to maximize efficacy while minimizing toxicity. Until mature survival data are available from these randomized trials, integration of ICI in MSS tumors remains investigational.

**Table 2:** Randomized Phase II/III Trials in pMMR/MSS (or unspecified) rectal cancer integrating immune checkpoint inhibitors. Abbreviations: EMVI+ extramural vascular invasion, MRF+ mesorectal fascia SCRT short course radiotherapy with 5 × 5 Gy, CRT chemoradiation, TNT total neoadjuvant therapy, FOLFOX fluorouracil, leucovorin, oxaliplatin, CAPOX capecitabine, oxaliplatin, TME total mesorectal excision, NOM nonoperative management, NAR neoadjuvant rectal score, SD standard deviation, c/pCR clinical/pathological complete response.

Trial	Phase	Patient Population	n	Interventions		Primary Endpoint
				Control Arm	Experimental Arm	
NRG-GI002 [24]	II	cT3–4 ≤ 5 cm from anal verge, ≤ 3 mm from MRF, cN2, not canditates for SSS	185	TNT with FOLFOX (4 months) > > capecitabine-based CRT > > TME	Same TNT + 6 x Pembrolizumab q3w (Start with CRT)	Mean NAR Score (SD) 14.08 (13.82) control vs. 11.53 (12.43) exp. *p* = 0.26
SPRING-01 [25]	II	cT3–4, cN+, EMVI+, MRF+ or enlarged lateral lymph nodes	98	TNT with SCRT >> 6 x CAPOX >> TME or NOM	Same TNT + 4 x Sintilimab q3w (Start with consolidation)	pCR Rate 32.7% vs. 59.2% *p* = 0.015
Xiao, Wei-Wie et al. [26]	II	cT3–4, cN+	134	TNT with 4 x CAPOX > > CRT >> TME or NOM	Same TNT + 4 x Sintilimab q3w (Start with CAPOX)	Total CR rate (pCR + cCR) 26.9% vs. 44.8% *p* = 0.031
POLARSTAR [27]	II	UICC Stage II/III (cT4b excluded), ≤ 10 cm from anal verge	186	capecitabine-based CRT > > TME	Same CRT + 3 x Tislelizumab q3w A: First week of CRT B: 2 weeks after CRT	pCR rate Control Arm A: 27.1% (*p* = 0.082) B: 32.7% (*p* = 0.019)
TORCH [28]	II	cT3–4, cN+, ≤ 12 cm from anal verge	121	TNT with SCRT > > 6 x CAPOX + Toripalimab > > TME or NOM	2 x CAPOX + Toripalimab > > SCRT > > 4 x CAPOX + Toripalimab > > TME or NOM	Total CR rate A: 56.5% B: 54.2% (both *p* < 0.001 vs predefined 25% after conventional TNT)
UNION [29]	III	cT3–4, cN+, ≤ 10 cm from anal verge	231	capecitabine-based CRT > > 2 x CAPOX > > TME > > 6 x CAPOX	SCRT > > 2 CAPOX + Camrelizumab > > TME > > 6 x CAPOX + Camrelizumab > > 17 x Camrelizumab	pCR rate 15.3% vs. 39.8% p < 0.001
STELLAR II [30]	II/III	cT3–4, cN+, ≤ 10 cm from anal verge	218	SCRT > > 4 x CAPOX > > TME or NOM > > 2 x CAPOX	Same TNT + Sintilimab with consolidation and adjuvant CAPOX	Total CR rate 25.0% vs. 45.5% *p* = 0.003

## 9 Conclusions

• NCCN-, ESMO-, an the German guidelines for rectal cancer now recommend TNT, particularly in the presence of at least one of the following risk factors: cT4, cN2, mrCRM+, EMVI+, or lateral lymph node involvement.
• TNT can be administered using either short-course radiotherapy (5 × 5 Gy) followed by consolidation chemotherapy, or fluorouracil-based CRT with induction/consolidation chemotherapy (CAPOX/FOLFOX). The optimal duration of induction/consolidation chemotherapy, however, remains unclear, given the wide variation across studies.
• The sequence CRT-consolidation therapy should be preferred, particularly when organ preservation is the goal for patients achieving (near) cCR.
• It remains unclear witch (subgroup of) patients should receive adjuvant therapy after TNT. Given that persistent tumor after intensive TNT may reflect resistant tumor biology, the clinical benefit of additional chemotherapy remains uncertain and may be outweighed by its toxicity.
• Current phase 3 clinical trials investigating TNT with the aim of organ preservation are addressing the following questions: 5 × 5 Gy vs. chemoradiotherapy (ACO/ARO/AIO-18.1); the use of mFOLFIRINOX vs. FOLFOX/CAPOX for consolidation chemotherapy (JANUS); and the inclusion of immune checkpoint inhibitors for patients with MSS tumors (STELLAR II).

## Author contribution

All authors have contributed equally to this manuscript.

## Funding

This research received no external funding.

## Declaration of competing interest

The authors declare that they have no competing financial interests or personal relationships that could have influenced this publication.
